# Giving Meaning to Non-Communicable Illness: Mixed-Method Research on Sense of Grip on Disease (SoGoD)

**DOI:** 10.3390/healthcare10071309

**Published:** 2022-07-14

**Authors:** Assunta Maiello, Ersilia Auriemma, Raffaele De Luca Picione, Daniela Pacella, Maria Francesca Freda

**Affiliations:** 1 Department of Humanities, University of Naples Federico II, 80138 Naples, Italy; assunta.maiello@unina.it (A.M.); fmfreda@unina.it (M.F.F.); 2 Department of Law, Giustino Forntunato University, 82100 Benevento, Italy; r.delucapicione@unifortunato.eu; 3 Department of Public Health, University of Naples Federico II, 80138 Naples, Italy; pacelladaniela@gmail.com

**Keywords:** chronic illness, narrative functions, primary care

## Abstract

When people receive a diagnosis of chronic or non-communicable disease, they need to reorganize their lives to understand and accommodate the changes associated with the new health condition. This reorganization, which involves the activation of a process through which meaning is given to the illness, could be fostered by narrative methods also in the context of Primary Care. The Sense of Grip on Disease (SoGoD) model intends to focus on the role of sense-meaning-making processes in the psychological adjustment to non-communicable illness, emphasizing the patients’ role in managing their own health condition. In this study, the authors propose a mixed-method research method which implies the adaptation of the narrative interview on the Sense of Grip on Disease. The interview was administered to 31 adults suffering from non-communicable diseases and has been analyzed with a theory-driven approach, which aims to explore the modalities of five narrative functions: organization of temporality, integration of illness, expression of emotions, social sharing and orientation to action. Through a Multiple Correspondence Analysis and a Cluster Analysis, the authors have identified two different ‘Grip Profiles’, called “Dynamic Profile” and “Compliant Profile”, representative of different degrees of flexibility, integration and adjustment to disease.

## 1. Introduction

### 1.1. Non-Communicable Disease and Primary Care

Chronic or non-communicable diseases, as the WHO [1] has defined them, are illnesses for which there is no definitive cure, but a range of various medical treatments and health behaviors useful for their management [2,3]. The spread of these illnesses has increased in the last two decades due to the increase in average life expectancy and recent medical progress, which have contributed to transforming illnesses with unfortunate outcomes, into chronic—and manageable—conditions [4,5]. The WHO [1] indicates that the four main groups of chronic or non-communicable diseases are: cardiovascular illness, cancer, diabetes and chronic respiratory disorders.

Although each of these diseases has its own specific characteristics, they all share a central aspect: they are pathologies that last a lifetime, and this results in specific medical, social and psychological needs [6,7]. Indeed, people with chronic or non-communicable diseases have to engage in the daily management of their conditions and, in order to better adjust to them, they have to understand and elaborate on the diagnosis, come to terms with their new identities as ill people and acquire psychological competences for coping with the new challenges and changes due to the illness [3,8,9,10]. These multiple psychological tasks should be accomplished with the help of physicians and healthcare workers in the primary care context; this represents the first level of healthcare assistance aimed at taking care of patients’ global needs, namely medical, psychological and social ones.

This is consistent with the biopsychosocial paradigm of health [11] and with the most relevant scientific frameworks about chronicity, such as Self-management, Empowerment, Health Engagement and the Chronic Care Model, which highlights the role of psychological aspects in the adjustment to disease [8,12,13,14,15,16,17,18].

Specifically, the Chronic Care Model [18] is an example of initiative medicine, namely a patient care model for the management of chronic diseases that does not “wait” for the patient to show up in hospital but goes to meet him by planning personalized interventions based on his health needs. In this approach, patients become an active part of their personalized care pathway, and the doctor-patient relationship addresses not only the medical but also the psychological, emotional and social dimensions that scientific literature considers so relevant in the adjustment to chronic diseases [19,20,21,22,23,24,25,26,27].

### 1.2. From Sense to Meaning: The Sense of Grip on Disease (SoGoD)

In this work, the authors intend to focus on the role of meaning in the adjustment to chronic or non-communicable diseases, from a narrative, semiotic and socio-constructivist perspective [28,29,30].

From this perspective, the onset of a chronic illness represents a critical experience, which disrupts the previous schemas of self and world, introduces a discontinuity in one’s own biography, and places the person in an intense affective state, characterized by confusion, uncertainty and fear of the future [31,32,33,34,35,36]. From the authors’ perspective, the first way of understanding this critical experience is the sense: this term refers to a primitive process of affective, visceral and bodily investment of the experience that cannot be expressed in words. In order to understand the illness and integrate it into one’s life story, the sense has to be transformed into a symbolic meaning, through a process that can be named *sense-meaning-making*. This process can unfold thanks to the construction of a narrative about the relationship between oneself, the disease experience and one’s own context. Indeed, for decades, narratives have been considered elective tools for the study of processes through which people make meaning of their critical experience of *turning points* [37,38]. Specifically, many scholars have found illness narratives the key to understanding the subjective and contextualized experience of ill people [39,40,41,42,43].

According to the relevance attributed to illness narratives, from the aforementioned semiotic and socio-constructivist perspectives, the authors have introduced in previous works a conceptual framework for understanding the sense-meaning-making process through illness narratives: the Sense of Grip on Disease (SoGoD) [44,45,46,47]. The word ‘*sense*’ is used in order to highlight the relevance of the sense-meaning-making process for the integration of illness into one’s experience. Concurrently, the word ‘*grip*’ is used in order to focus on the development of psychological competencies needed for the management of the disease and of the personal, relational and contextual changes that it entails. From the authors’ perspective, reaching a flexible grip on disease means having the competencies to emotionally and cognitively understand it, and being personally involved in its management [44,45].

This conceptual framework adopts a functional perspective on narrative, thus, it focuses on the transformative function of narrative through which the affective and primitive sense of a critical experience can be transformed into meaning. This transformative function can be better understood by breaking it down into more specific functions. Specifically, the authors have identified five key narrative functions [44,45].

1. *Organization of Temporality*. This refers to the temporal articulation of disease experience [48,49,50,51]. Through this function, it is possible to frame the disease in the time of a person’s existence [52,53,54], and to recognize turning points and differences between the time before and after the diagnosis.
2. *Integration of disease*. This refers to the process of construction of a subjective theory through which patients give meaning to the experience of disease, consistent with personal values, norms and aims [37,55]. From her own subjective theory, a person can integrate the disease into her story in different ways [10].
3. *Expression of emotions*. This refers to the process through which it is possible to express and differentiate emotions and feelings connected to illness [56,57].
4. *Social sharing*. This refers to the way by which people narrate their disease to other people, and to the sense they give to this social sharing. The narrative process works as a tool for sharing feelings and points of view and for gaining social support [40,58].
5. *Orientation to action*. This refers to narrative agency, namely the process by which people actively recognize their role in the process of management of their chronic condition [50].

Each function can work at different levels of integration, discretization and flexibility: authors identify these different levels with the term *sensemaking modalities*. Through an ad hoc semi-structured narrative interview, the model of SoGoD intends to capture the different modalities of each function, namely the different modalities through which people give meaning to their illness experience. In previous work, the authors have explored the Sense of Grip of parents of children affected by Hereditary Angioedema [44], of adult people suffering from Hereditary Angioedema [45] and of a wider sample of children suffering from different chronic conditions [46,59]. Such narrative interviews have always been shown to opportunely solicit in chronic patients a subjective presentation of their illness experiences from many perspectives.

In this work, the authors intend to present mixed-method research on Sense of Grip on Disease with adult patients suffering from non-communicable diseases, in order to evaluate whether the Sense of Grip on Disease model can be used to understand the experience of these patients. The authors aim to identify different Grip Profiles, based on the narratives emerging from the Narrative Semi-Structured Interview on Sense of Grip on Disease. The results suggest the presence of two main Grip Profiles.

## 2. Materials and Methods

### 2.1. Aims and Design

The aims of the study are to highlight, using a mixed-method design, the different ways by which people suffering from non-communicable diseases give meaning to their health conditions. The authors chose to use a mixed-method research design as it allows us to not give up on grasping the subjectivity of the individual’s experience, although the goal is to identify prototypical models of adaptation to the disease.

The qualitative coding of the interviews allows us to grasp coherently, with the theoretical model, the way in which each narrative function is expressed in the narration of each participant; the multiple correspondence analysis allows us to statistically understand the main dimensions of the theoretical model, as they emerge from the specificity of the data.

The cluster analysis shows how these dimensions aggregate in clusters. In summary, the mixed-method used here allows us to understand the way in which the different modalities of the different narrative functions move together and shape “typical” ways of giving meaning to the disease and adapting to it. In addition, the research strategy included statistical analyses to evaluate the differences, with respect to some variables, between the clusters that emerged.

Specifically, in this work the authors intend to:

1. Explore, through an adaptation of the Narrative Semi-Structured Interview on Sense on Grip on Disease [60] the principal dimensions of Sense of Grip on Disease in people affected by different non-communicable diseases;
2. Identify different Grip Profiles, representative of different ways to adjust to disease and integrate it into one’s own experience;
3. Study the association between the Grip Profiles and both the quality of healthcare assistance and patient engagement.

### 2.2. Participants

The research has been conducted with a convenience sample of people affected by non-communicable diseases. The eligibility criteria were:

- Aged between 34 and 75 years old (this range includes adults, late adults and young elderly);
- Having one or more chronic conditions included in the four main groups of non-communicable diseases [1]: cardiovascular illness, cancer, diabetes and chronic respiratory disorders.

The sample obtained is composed of 31 people (19 M, 12 F; medium age: 57, 45 ± 9 and 23) suffering from non-communicable diseases. The mean time from diagnosis is 13, 64 ± 10 and 17 years. Within this sample, 11 people have one disease (5 hypertension, 2 heart failure, 1 diabetes, 1 chronic respiratory disorder and 1 cancer) and 20 people have 2 or more chronic diseases. A summary table about the participants is reported below (Table 1).

### 2.3. Instruments

#### 2.3.1. Narrative Semi-Structured Interview on Sense on Grip on Disease 

The interview is an adaptation of the Narrative Semi-Structured Interview for people with Hereditary Angioedema [45,60].

It is composed of 13 questions about the disease experience, and aims at exploring the five narrative functions, namely the way by which people:

1. Organize illness experience with respect to past, present and future (organization of temporality);
2. Come to terms with illness and integrate it into their horizon of values, representations and aims (integration of disease);
3. Express the emotions and feelings related to illness (expression of emotions);
4. Talk about their disease experience and share it with relatives, friends, colleagues and/or healthcare workers (social sharing);
5. Come to terms with the daily management of their own condition, for example taking pills, doing exercise and making decisions of activities on their own initiatives, etc., (orientation to action).

The interview was constructed to capture how people relate to the chronic nature of their illness. In fact, questions have been built to be used with people with a variety of chronic or non-communicable conditions, as they explore aspects common to all people who have a disease that lasts over time, such as symptom management, diagnosis and changes in social relationships.

In this work, the interview also involves questions about how the COVID-19 pandemic has impacted people’s disease experiences. The interview is fully reported in Appendix A.

#### 2.3.2. Patient Health Engagement-Scale 

The Patient Health Engagement-Scale [61] is a self-reported questionnaire that measures the state of a patient’s engagement in her care path. Health engagement is a process that evolves through four phases: *black out* (the person is unable to elaborate their illness diagnosis), *alert* (the person has begun to process the diagnosis but is still scared of each abnormal signal of their body), *adhesion* (the person has integrated their new identity as an ill person), *eudaimonic project* (the person is able to project themselves into the future together with the disease and despite of it). As the patient evolves in the engagement process, they will be more able to take ownership of their care process, while in the early stages they will have a greater need to delegate decisions about their health to the doctors.

The scale consists of 5 items that measure the cognitive, emotional and behavioral aspects of engagement. The items are rated on a 7-point Likert scale and the final score is the median of the scores obtained. The score places the patient in one of the 4 phases previously described.

#### 2.3.3. Patient Assessment of Chronic Illness Care (PACIC-20) 

PACIC-20 [62] is a self-reported questionnaire that measures the patient-assessed quality of care received from general practitioners in the past six months. It is composed of 20 items divided into 5 sub-scales: patient activation (3 items), delivery system/practice design (3 items), goal setting/tailoring (5 items), problem-solving/contextual counseling (4 items) and follow-up/coordination (5 items). The items are rated on a five-point Likert scale, ranging from 1 (never) to 5 (always), with higher scores indicating a better patient-assessed quality of care and greater alignment with the Chronic Care Model.

#### 2.3.4. SF-12 Health Survey 

SF-12 [63,64] is a self-reported questionnaire that measures quality of life related to health in the past month. It is composed of 12 items divided into two subscales: the Mental Component Scale (MCS) which measures the psychological dimension of quality of life, and the Physical Component Scale (PCS) which measures the physical dimension of quality of life. Higher scores in each subscale indicate a better quality of life related to mental and/or physical dimensions.

### 2.4. Procedure

The research has been conducted thanks to the involvement of six general practitioners from two Territorial Functional Aggregations in Naples. Each practitioner has proposed participation in the study to patients suffering from one of the four main groups of non-communicable diseases.

Participation in the study involved the completion of a questionnaire containing different scales for measuring psychological variables related to adjustment to illness, and participation in a narrative interview. The questionnaire was sent to patients by general practitioners via the Google Modules platform. The interviews were collected by telephone by two psychologists trained in its administration and analysis; interviews were audio-recorded with the consent of the participants and subsequently transcribed verbatim.

Each participant signed an informed consent form for participation in the study and a document for the protection of privacy in accordance with the GDPR EU 2016/679, D.L. 101/2018. The study was conducted in accordance with the Declaration of Helsinki and approved by the Ethics Committee of Psychological Research of the Department of Humanities of the Federico II University of Naples (Prot. n. 24/2021).

### 2.5. Methods of Analysis of Narrative Corpus

#### 2.5.1. Qualitative Analysis of Narratives

The coding of the narrative corpus was conducted through a theory-driven approach, starting from the functional perspective of the narrative [44,46,65,66]. The analysis of the narrative functions was conducted by identifying, in the narratives, the representative extracts of one of the five narrative functions described in the previous paragraph: temporal articulation, integration of the disease, expression of emotions, social sharing and orientation to action [44,45,46,67].

The coding grid is schematically reported in Table 2, with a code assigned to each modality for the Multiple Correspondence Analysis. The authors will discuss the declination of each modality in the results section, with some representative narrative extracts from the interviews of this study for each modality. The narrative analysis was carried out by three independent researchers trained in the analysis of the interviews and in the use of the narrative functions coding grid. In cases where there was a discrepancy in the coding, a group discussion took place until an agreement was reached between the researchers.

#### 2.5.2. Quantitative Analysis of Narratives

Based on the coding of the narrative interviews, a Multiple Correspondence Analysis (MCA) was performed, in order to identify the main dimensions of the Sense of Grip on Disease. Indeed, the MCA shows the association between the categorical variables considered through the extraction of the main dimensions. A hierarchical clustering model was then applied to the main dimensions of Sense of Grip allowing the identification of co-occurrence clusters: each cluster identifies a group of subjects with similar characteristics, in this case, with similar modalities of narrative functions. The statistical significance of the modalities of each variable within the clusters was determined with a v-test (equivalent to a chi-square test) applied to the modalities, in order to evaluate the difference between the frequencies of the modalities in the cluster compared to their frequencies in the total dataset.

### 2.6. Statistical Analysis for Association between Clusters and Psychological Variables

Results of PACIC, SF-12 and PHE-S have been added as additional variables to the MCA model, in order to identify the difference between the two clusters relating to patients’ engagement and patient-assessed quality of care. The difference between the distributions of scores within each cluster was evaluated using non-parametric tests (Kruskal–Wallis for quantitative variables and Fisher’s exact test for categorical variables).

It was considered a significance level α = 0.05. Analyses were performed with IBM SPSS Statistics for Windows, version 26.0 (Armonk, NY, USA) and with R, version 4.0.3 (Vienna, Austria).

## 3. Results

### 3.1. The Declination of Narrative Sensemaking Modalities

For each modality, it will be reported the number and the percentage of narratives codified as representative of it. Then, each modality will be described with the help of some representative extracts from the interviews of patients.

1. Organization of temporality
  - *Absence*: 5 (16.13%). This modality refers to a temporal framework in which there is no difference between the time before and after the onset of the disease. This is typical of the narratives in which the disease is represented as irrelevant to the patients’ life stories: it is not considered a turning point, so it has not the power to mark the time of one self’s existence.
    *«Nothing has changed, really nothing. It’s everything like before»*
  - *Crystallization*: 12 (38.71%). This modality refers to narratives in which the time appears to be blocked and is not allowed to flow. Specifically, the temporal framework is frozen at the time of diagnosis, or it is crystalized in an eternal and precarious present marked by the necessities connected to disease management. This modality is typical of narratives in which there are many chronological references to the time of medications and/or medical checks: the time of life totally coincides with the time of illness.
    *«I can’t make long-term plans, because my life is punctuated by periodic and obligatory medical checks every 6 or 12 months. I have to deal with the unpredictability of disease, I don’t know what… Anyway, I live, I try not to think about it, but I still live in a precarious situation, I am unable to plan my existence as I did before»*
  - *Transformation*: 14 (45.16%). This modality refers to a temporal framework that evolves following the evolving flow of experience. It is typical of the narratives in which it is marked a difference between the time before and after the onset of the illness, or in which the experience of the illness itself seems to evolve during the time.
    *«The more the years go by, the more I feel calm, because it means that you can manage it, you can control it, obviously with medications. All in all, the more time has passed, the more reassured I am»*
2. Integration of disease
  - *Conflict*: 2 (6.45%). This modality refers to a struggle between the self and the disease. It is typical of the narratives that express a difficulty to elaborate on the diagnosis, understand the illness and integrate it into one’s story. The illness has been represented as an enemy or a part of life that is impossible to accept because it has destroyed the previous versions of the self and of the world.
    *«There is no remedy, here, there is no remedy, it cannot be, it is not something that can be solved... and then this is it, it is anger, anger every day, every day is anger, more than anything else it is anger... I’m angry with the disease, that’s it, that’s it»*
  - *Tolerance*: 17 (54.84%). This modality refers to an acceptance of the illness characterized by the lack of a subjective meaning for the experience. It is typical of narratives in which the disease is represented as something that happens for no particular reason and that has to be accepted without asking what it could mean for oneself.
    *«The problem is there, it is there, it has come and we keep it. We can do very little, it would have been better if he hadn’t come, but he did»*
  - *Coexistence*: 12 (38.71%). This modality refers to a full integration of the disease into one’s own life story. It is typical of the narratives in which the disease has been represented as a part of oneself, although not the most relevant part. The narrative expresses a good elaboration of the diagnosis and the ability to understand the changes imposed by the pathology and the negative sides of it, without feeling overwhelmed by them.
    *«I mean, actually for me it’s a stress, but it’s a stress I’ve lived all my life, it’s a stress… I mean, for sure it isn’t totally positive, but I live it as... I see my life as a crossword puzzle, do you know the crossword puzzle? In the cross puzzle there are words that have to fit together, so I see all the problems we have as a challenge to find the solution»*
3. Expression of emotions
  - *Vagueness* (17 (54.84%). This modality considers the affective sphere (moods, affects, emotions and feelings) as little differentiated, confused and often totalizing. This way shows a lack of contextualization, and a difficulty in expressing more detailed information about the emotional features of one’s own experience (e.g., “I feel bad” without explaining why or when). Throughout the narrative, we find expressive poverty from an emotional point of view and a very small range of emotions.
    *«It’s anger, anger…… most of all it’s anger. I’m angry with disease… everyday it’s anger»*
  - *Differentiation*: 22 (45.16%). This modality refers to the expression of affects, feelings and emotions related to the illness, often linked to specific episodes or situations. The patient is able to consider different emotional nuances and is capable of having and using a wider vocabulary of possible descriptions for affective states, emotions related to different circumstances, and different feelings that bind them to other people.
    *«I am worried, but in some moments, I am also serene. I mean, on the one hand, I’m worried, but I don’t feel it as a burden [...] I’m worried about the disease, not because I have to go back to chemo or I may have a relapse, but because I’m afraid of feeling really bad. But even if I am worried, I always remain optimistic, I did not despair, I always thought that I would come out of it, even with difficulty»*
4. Orientation to action
  - *Executive*: 22 (70.97%). This modality refers to a rigid, schematic and non-personalized management of the disease. The agency is often delegated to someone else, typically to physicians and/or family members, sometimes with a feeling of being trapped by the obligations associated with the disease (e.g., Taking pills or following a diet).
    *«My wife tries to control me, to help me, sometimes I get angry because she controls me on food choices, if she were not here with me, I would already be dead»*
  - *Flexible*: 9 (29.03%). This modality refers to the assumption of responsibility and to the competence of decision-making related to illness management. It is typical of narratives that express different ways of illness management related to different situations: the therapeutic adherence is contextualized, and the patient is personally engaged in the management of her condition.
    *«I do things more in the morning than in the afternoon, because I feel very tired in the afternoon. For example, if I wanted to clean the house in the afternoon, I couldn’t, so in the morning I get up at 7 and I do it, while in the afternoon I begin to get tired and then at 21:30 I go to bed, otherwise I can’t do anything»*
  - *Limiting*: 0 (0%). This modality refers to the limitation of one’s daily activities out of fear of the disease and/or its consequences.
5. Social sharing
  - *Loneliness*: 10 (32.26%). This modality refers to the absence of social sharing of difficulties, emotions and thoughts about the illness. It is typical of narratives in which people say that the disease is not a subject of conversation with friends, family or practitioners, or of narratives in which appears a difficulty in sharing with others the new identity of the ill person.
    *«I don’t talk about it with anyone… no…no…anyone, absolutely anyone»*
  - *Interchange*: 16 (51.61%). This modality refers to the use of social conversation for asking or communicating information about the disease. It is typical of narratives in which others are represented as able to provide concrete support, but their presence or absence is not so relevant in terms of emotional relief or feelings of being understood.
    *«Talking about it doesn’t change things so much… of course I feel supported if, for example, I have to do a checkup and my son come with me, but then it doesn’t change me very much if for one reason or another he’s not there»*
  - *Sharing*: 5 (16.13%). This modality refers to the use of social conversation for sharing emotions, feelings and points of view about the disease. It is typical of narratives in which the narrator shares with others the meaning he has attributed to illness and in which others are represented as significant for the emotional support they provide and for the ability to understand the narrator’s needs.
    *«Sometimes I talk about it with friends, as I told you I am a head teacher, and I also talk about it with some more sensitive teachers who understand, perceive the diversity of my gaze [...] it makes me feel good talking about it, of course, above all when I feel listened to. »*

### 3.2. Results Multiple Correspondence Analysis

The application of MCA showed a good fit of the coding grid to the data. The first two dimensions extracted explains 48.2% of the variance.

**Figure 1:**
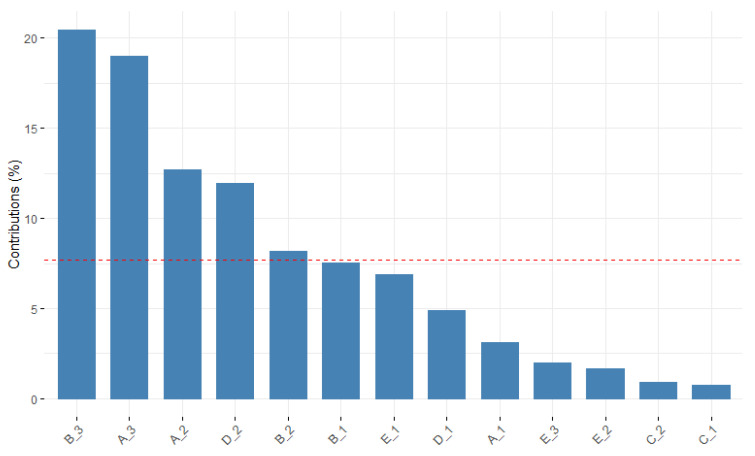
MCA_ Dimension 1.

Figure 1 shows the contribution of each variable—in this case, of each modality—to the first dimension. The red line represents the mean contribution expected, and the modalities that exceed this threshold are considered representative of this dimension. So, the modalities that contribute most to the first dimension are:

- B_3 (integration–coexistence)
- A_3 (organization of temporality–transformation)
- D_2 (orientation to action-flexible)
- B_2 (integration–tolerance)

**Figure 2:**
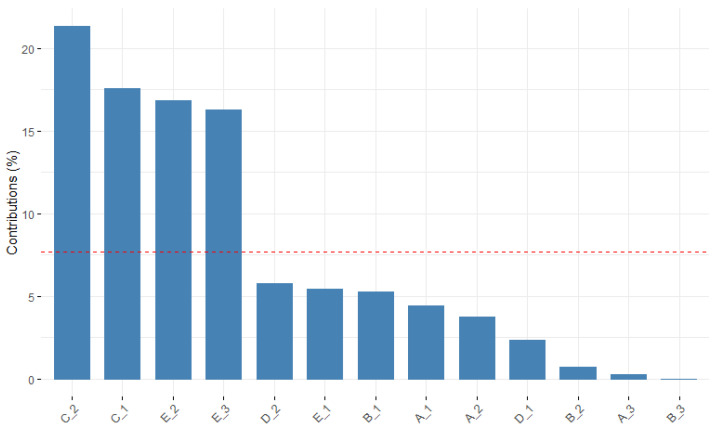
MCA_ Dimension 2.

Figure 2 shows the contribution of each modality to the second dimension. The modalities that contribute most to the second dimension are:

- C_2 (expression of emotions-vagueness)
- C_1 (expression of emotions–differentiation)
- E_2 (social sharing–interchange)
- E_3 (social sharing–sharing)

**Figure 3:**
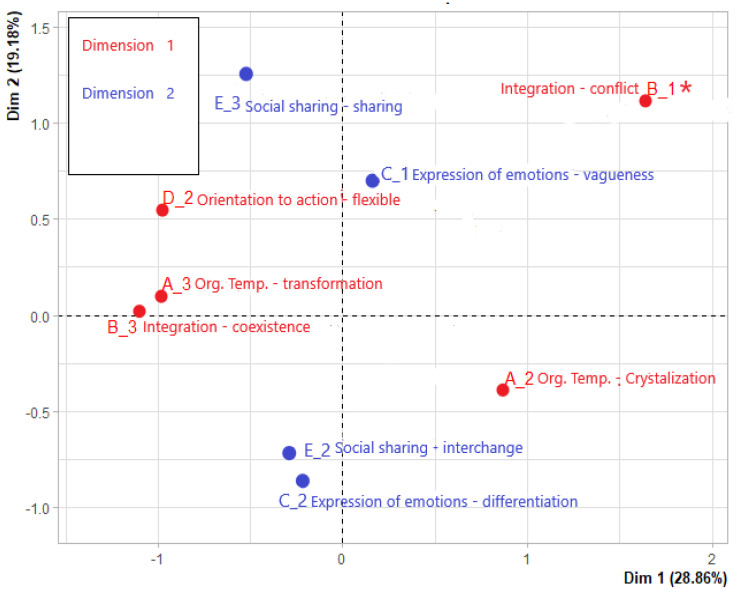
Factor map with modalities.

As the Figure 3 shows, the first dimension is composed of the modalities of the following narrative functions: integration of disease, organization of temporality, and orientation to action. The second dimension, on the other hand, is made up of modalities of the narrative functions of social sharing and expression of emotions.

Specifically, on the axis corresponding to the first dimension, the flexible, transformation and coexistence modalities are opposed to the crystallization and conflict modalities; the conflict modality is just below the level of significance for the first dimension (for this reason it is marked with an asterisk) but its position in the factor map is particularly consistent with the model of Sense of Grip on Disease.

On the axis corresponding to the second dimension, the vagueness and sharing modalities are opposed to the interchange and differentiation ones. The interpretation of these results in the light of the model of Sense of Grip on Disease will be presented in the discussions.

### 3.3. Results of Cluster Analysis

Starting from the analysis of the first dimension identified since it was prevalent, a hierarchical cluster analysis was performed. From its dendrogram, two main clusters emerged, which identify groups of individuals with similar characteristics (in this case, with similar modalities of narrative functions).

The *p*-values of the modalities whose presence or absence in a cluster was significantly different from the overall dataset are shown in the tables below, alongside the v test, whose sign of the raw value indicates whether there is a greater presence or absence of the modality in the cluster than in the dataset.

As Table 3 shows, Cluster 1 is significantly associated with modalities A_3, B_3, D_2, D_1, B_2, A_2.

Specifically, it is characterized from high frequency of:

- A_3 (organization of temporality–*transformation*)
- B_3 (integration-*coexistence*)
- D_2 (orientation to action-*flexible*)

and low frequency of:

- D_1 (orientation to action-*executive*)
- B_2 (integration-*tolerance*)
- A_2 (organization of temporality-*crystallization*).

The factors are ordered from the most common to the rarest.

As Table 4 shows, Cluster 2 is significantly associated with modalities: A_2, B_2, D_1, D_2, B_3, A_3. Specifically, it is characterized by high frequency of:

- A_2 (organization of temporality-*crystallization*)
- B_2 (integration-*tolerance*)
- D_1 (orientation to action-*executive*)

and low frequency of:

- D_2 (orientation to action-*flexible*),
- B_3 (integration-*coexistence*)
- A_3 (organization of temporality-*transformation*)

Factors are ordered from the most common to the rarest.

### 3.4. Results of Statystical Analysis for Association between Clusters and Psychological Variables

As Table 5 shows, the results of Kruskal–Wallis test shows that mean scores of PHE-S and of PACIC-Patient activations are significant higher in cluster 1 than in cluster 2 (*p* = 0.027; *p* = 0.034). There are no other statistically significant differences between clusters.

## 4. Discussion

### 4.1. The Articulation of Narrative Functions

Results show a good adaptation of the Coding Grid, developed in a previous study with adult patients affected by Hereditary Angioedema [45] to narratives of patients affected by different non-communicable conditions. Furthermore, in previous research [46] the authors already developed and verified the extension of the use of the interview and the coding grid for the parents of chronic young patients (affected by several chronic illnesses). In the current study as well, the interview and the coding grid appear as congruent tools to explore the experience of illness in conditions of chronicity. The only modality that was present in the grid but not identified in any of the narratives is the *limiting* modality of orientation to action.

This modality generally appears in the narratives of people who severely limit their daily actions for fear that these could have negative effects on the disease. Probably, it was often present in the narratives of people with HAE because of the unpredictability of the disease which makes it hard for patients to understand when and how to cope with the symptomatology. However, people with non-communicable diseases usually have enough information to understand the characteristics of their disease and the behaviors required to manage it without feeling the necessity to limit their daily lives.

### 4.2. The Main Dimensions of Sense of Grip

Using MCA it was possible to identify the main dimensions within which the narratives differ: these can be considered the main dimensions of the Sense of Grip on Disease. The coding system has proved effective since the two dimensions explain more than 48% of the total variability, this means that the variables identified for the analysis measure relevant and different aspects and are able to explain the differences between the units of analysis (in our case the narratives).

Results of MCA show that the two dimensions identify different aspects of Sense of Grip on Disease. Indeed, the first dimension deals with cognitive and reflexive aspects of the relationship to the disease, while the second dimension concerns the competence to recognize and express this relationship, also considering the social context, affects, emotions and feelings related to the disease. Consistent with the model of Sense of Grip on Disease, along the first dimension a contrast emerges between: (a) reflexive processes, which result in a good elaboration of the diagnosis (integration of the disease in everyday life, organization of flexible management strategies and representation of the disease as a process that changes over time); and (b) reflexive processes, which result in a conflictual and static relationship with the disease. Along the second dimension, a contrast emerges between good emotional differentiation and a vague and generalizing quality of affects; in the same way, a contrast emerges between the use of the narrative of illness as an exchange of information and its use as a sharing of one’s experiences.

### 4.3. The Grip Profiles

The cluster analysis identifies two main clusters that aggregate individuals whose narratives express, within each function, similar modalities of articulation of the narrative process. Each cluster has been interpreted in terms of the Grip Profile.

**Cluster 1—entitled *Dynamic Profile*.** It is characterized by these modalities (ordered from the most to the less significant): for the function and organization of temporality, the modality defined “transformation”; for the function of integration, the modality defined “coexistence”; and for the function of orientation to actions, the modality defined “flexible”. This cluster has been defined—consistent with all previous studies [44,45,46]—as *Dynamic Profile*, since the narratives that belong to it have in common dynamic, flexible and complex processes of sense-meaning-making. These narratives highlight a moving temporal trajectory: the disease is represented as a relevant biographical event, which has transformed life, and which continuously changes over time, concurrent with changes in symptoms, life cycles, needs and life contexts. The narrative process builds links between the past, the present, and sometimes, the future, and within this complex temporal perspective, it is possible to integrate the disease as a significant but not cumbersome aspect of life. Indeed, although it is possible to recognize the negative side of being ill, it is also possible to recognize one’s own resources. In fact, the narratives that belong to this cluster seem to be characterized by flexible disease management processes, that is, the ability to mediate between the needs and constraints connected to the pathology and one’s own subjective needs and personal resources.

**Cluster 2—entitled *Compliant Profile*.** It is characterized by these modalities (ordered from the most to the less significant): for the function of organization and temporality, the modality defined “crystallization”; for the function of integration, the modality defined “tolerance”; and for the function of orientation to actions, the modality defined “executive”. This cluster has been defined as *Compliant Profile* since the narratives that belong to it seem to be united by schematic and stereotyped sense-meaning-making processes, oriented towards the assimilation of disease and adherence to medical prescriptions. In particular, narratives are characterized by a rigid temporal trajectory, marked by medical checks and treatments, and into which the disease is collocated as a stationary event, rather than as a process that evolves over time. Illness is an aspect of life that exists and that must be accepted/tolerated, but it is not fully understood, elaborated and integrated into one’s story, even if there are no explicit aspects of the conflict. The relationship between the self and the disease seems not to be affectively connoted and is based on the control of symptoms through medications or lifestyle.

### 4.4. Grid Profiles and Involvement of Patient

Results show that both patient engagement and patient activation are significantly higher in the Dynamic Profile than in the Compliant Profile. This is consistent with the Sense of Grip of Disease since this model suggests that the more flexible, dynamic and complex the sense-meaning-making process, the more emotionally and cognitive involved in one’s own health condition is the person. Indeed, if a person is able to represent the illness as an integrated part of their life, that can change over time and can be managed with flexible and diversified strategies in line with personal needs; it is likely they have integrated the new identity of ill person and are ready to assume ownership of the management of their health condition [68,69,70]. On the contrary, if a person is not able to recognize the critical aspects of the disease as well as its processual nature, probably they are more inclined to utilize rigid coping strategies and to delegate decisions related to their health condition to doctors.

Surprisingly, quality of life seems not to be associated with the quality of the sense-meaning-making processes, contrary to what has emerged in the study with patients affected by hereditary angioedema [45]. It seems that in our sample the two clusters that emerged are representative of two different ways of experiencing the disease; one more based on integration and the other more based on adhesion. These differences, however, seem to have an impact on the level of participation in the healthcare relationship and on the quality of engagement in self-care, but not on the perception of the quality of one’s health condition.

## 5. Conclusions

This study highlights the efficacy of the model of Sense of Grip on Disease (SoGoD) in exploring the sense-meaning-making processes which regulate the adaptation of people with non-communicable diseases to their medical conditions.

The results show two main dimensions of Sense of Grip: one concerning the cognitive processing of the disease, and one concerning emotional processing and social sharing.

The two Grip Profiles that emerged in this study deal only with the first dimension, so they represent two different levels of the reflexive and cognitive process of adjustment to illness, with the Dynamic Profile being more capable of understanding the illness and integrating it into daily life with personalized and flexible management strategies. This is consistent with the results of PHE-S and PACIC. Indeed, the levels of the patient’s involvement are significantly higher in the group of people with a Dynamic Profile than in that with Compliant Profile.

Despite the limits of this study, primarily due to the low sample size, results suggest that promoting Sense of Grip could encourage patients to be more engaged in the management of their conditions, in accordance with the principles of the main scientific frameworks briefly discussed in the introduction of this work. Specifically, in the context of primary care and in collaboration with general practitioners, it would be useful to design psychological interventions aimed at promoting more complex, flexible and differentiated sense-meaning-making processes. The narrative interview on the SoGoD could thus be used for clinical purposes: it allows us to identify the Grip Profile, therefore it would help us to plan psychological interventions consistent with specific patients’ needs and competence.

Future trajectories of development of this research concern finding a more detailed understanding of the relationship between the sense-meaning-making process and quality of life in people with chronic conditions; achieving a more detailed understanding of the emotional processes in the elaboration of one’s own illness; extending the application of the SoGoD Interview with a larger sample and wider range of chronic illnesses; deepening of the processes of construction of the Sense of Grip occurring in the time in order to offer relevant and useful indication for the context of the psychology of health, and psychological clinical interventions.

## Figures and Tables

**Table 1:** Participants.

Variable		Participants (n = 31)
** Age**		57.45 (±9.23)
** Years from Diagnosis**		13.64 (±10.17)
** Sex**		
	** Female**	12 (39%)
	** Male**	19 (61%)
** Comorbidity with other**	** Yes**	20 (64.5%)
** chronic conditions**	** No**	11 (35.5%)

**Table 2:** Narrative sensemaking modalities coding grid.

Narrative Function	Modalities
**Organization of temporality**	Absence A1 Crystalization A2 Transformation A3
**Integration of disease**	Conflict B1 Tolerance B2 Coexistence B3
**Expression of emotions**	Vagueness C1 Differentiation C2
**Orientation to action**	Executive D1 Flexible D2 Limiting D3
**Social sharing**	Loneliness E1 Interchange E2 Sharing E3

**Table 3:** Cluster 1.

Cluster 1				
	Cla/Mod	Mod/Cla	*p*.Value	v.Test
**A = A_3**	100.00000	93.33333	<0.001	5.429334
**B = B_3**	100.00000	80.00000	<0.001	4.655995
**D = D_2**	88.88889	53,33333	<0.001	2.770115
**D = D_1**	31.81818	46,66667	<0.001	−2.770115
**B = B_2**	17.64706	20.00000	<0.001	−3.696399
**A = A_2**	0.00000	0.00000	<0.001	−4.361849

**Table 4:** Cluster 2.

Cluster 2				
	Cla/Mod	Mod/Cla	*p*.Value	v.Test
**A = A_2**	100.00000	75.00	<0.001	4.361849
**B = B_2**	82.35294	87.50	<0.001	3.696399
**D = D_1**	68.18182	93.75	<0.001	2.770115
**D = D_2**	11.11111	6.25	<0.001	−2.770115
**B = B_3**	0.00000	0.00	<0.001	−4.655995
**A = A_3**	0.00000	0.00	<0.001	−5.429334

**Table 5:** Differences between clusters.

	Cluster 1 n = 15	Cluster 2 n = 16	*p*.Value
**PACIC—Patient activation**	**3.04 (1.13)**	**2.21 (0.94)**	**0.034**
**PACIC—Delivery system/practice design**	3.67 (1.30)	3.12 (1.29)	0.254
**PACIC—Goal setting/tailoring**	2.63 (1.07)	2.21 (0.93)	0.261
**PACIC—Problem solving/contextual counselling**	2.63 (1.46)	2.28 (1.27)	0.480
**PACIC—Follow-up/coordination**	2.43 (1.37)	1.80 (0.70)	0.128
**PHE-S**	**3.33 (0.62)**	**2.75 (0.77)**	**0.027**
**PCS-12**	49 (11)	49 (7)	0.80
**MCS-12**	55 (8)	53 (9)	0.62

## Data Availability

Not applicable.

## Appendix A

1. When have you discovered your medical problems for the first time?
2. Do you remember what did you feel and what did you think when you received the diagnosis?
3. Could you please tell me two words that describe how do you live your illness’ experience?
4. You told me [first word said by interviewed], could you please tell me a specific episode that could help me to understand what do you mean when you say [first word said by interviewed]?
5. You told me [second word said by interviewed], could you please tell me a specific episode that could help me to understand what do you mean when you say [second word said by interviewed]?
6. How do you think your disease controls or affects your life?
7. How do you usually manage your medical condition in everyday life?
8. If you think about your experience, how do your symptoms vary? Do you think these variations in symptoms are linked to something?
9. Are you taking drug therapy? If yes, how is your relationship with medicines?
10. Do you think that there is someone or something particularly useful for the daily management of your disease?
11. Do you usually talk about your disease with someone? How do you talk about it and how does it feel when you talk about it?
12. Has your way of thinking about the disease changed over time?
13. How do you think that COVID-19 pandemic has affected your illness’ experience?
14. What do you bring with you from your illness’ experience?
